# Comparison of our self-designed rotary self-locking intramedullary nail and interlocking intramedullary nail in the treatment of long bone fractures

**DOI:** 10.1186/1749-799X-9-47

**Published:** 2014-07-21

**Authors:** Bailian Liu, Ying Xiong, Hong Deng, Shao Gu, Fu Jia, Qunhui Li, Daxing Wang, Xuewen Gan, Wei Liu

**Affiliations:** 1 Department of Orthopaedics, Yan’an Hospital, No. 245 Renmin East Road, Kunming, Yunnan 650051, China; 2 Emergency Department, Yan’an Hospital, Kunming, Yunnan 650051, China

**Keywords:** Rotary self-locking intramedullary nail, Interlocking intramedullary nail, Retrospective analysis, Long bone fractures

## Abstract

**Objective:**

The purpose of this study is to compare the clinical effects of our self-designed rotary self-locking intramedullary nail (RSIN) and interlocking intramedullary nail (IIN) for long bone fractures.

**Methods:**

A retrospective study was performed in 1,704 patients who suffered bone fractures and underwent RSIN or IIN operation in our hospital between March 1999 and March 2013, including 494 with femoral fractures, 572 with humeral fractures, and 638 with tibial fractures. Among them, 634 patients were followed up for more than 1 year. The operative time, intraoperative blood loss, postoperative complications, healing rate, and the excellent and good rate of functional recovery were compared between two groups.

**Results:**

Compared with IIN group, RSIN group exhibited significantly shorter operative time and less intraoperative blood loss no matter for humeral, femoral, or tibial fractures (all *p* < 0.001). The healing rate in patients with more than 1 year follow-up was significantly higher in RSIN group for femoral and tibial fractures (both *p* < 0.05). In RSIN group, no nail breakage or loosening occurred, but radial nerve injury and incision infection were respectively observed in one patient with humeral fracture. In IIN group, nail breakage or loosening occurred in 7 patients with femoral fractures and 16 patients with tibial fractures, radial nerve injury was observed in 8 patients with humeral fractures, and incision infection was present in 2 patients with humeral fractures and 1 patient with femoral fracture. The complication rate of IIN group was significantly higher than that of RSIN group (*p* < 0.05). However, there were no significant differences in the excellent and good rate of shoulder, elbow, knee, and ankle joint functional recovery between RSIN group and IIN group.

**Conclusion:**

RSIN may be a reliable and practical alternative method for the treatment of long bone fractures.

## Background

Long bone fractures, including tibial, femoral, and humeral fractures, are common traumatic injuries, accounting for approximately 4% of emergency department visits in the USA every year [1]. The management of long bone fractures remains challenging and often controversial. Recently, closed or open reduction and internal fixation with interlocking intramedullary nailing (IIN) has been recommended as a standard approach for treatment of long bone fractures because it has the advantages of fixation stability, anti-rotation, and anti-contraction, contributing to a high healing rate and a low incidence of complications [2-4]. However, the use of interlocked nail also has some disadvantages: it is a demanding step for accurate insertion of the distal locking screws, leading to increased radiation exposure and delayed operative time [5,6]; rotator cuff tear and shoulder impingement are easily caused for fixation of humeral fractures, resulting in shoulder pain and shoulder function dysfunction [7]. Because of excessive bending stress in femur and tibia, the IIN nail has the potential risk of breakage for fixation of tibial and femoral fractures [8,9].

To overcome the drawbacks of IIN for clinical fixation of long bone fractures, from 1995 to 1998, our team developed self-locking intramedullary nails (RSIN) for fixation of the humeral, femoral and tibial fractures. RSIN can be directly screwed into the medullary cavity with the side-locking tag to achieve anti-rotation. The purpose of present study was to retrospectively review the clinical and therapeutic outcomes of IIN and our RSIN for treatment of long bone fractures.

## Methods

### Patients

Totally, 1,704 patients (approved by ethics committee of Yan’an Hospital) suffering humeral (*n* = 494), femoral (*n* = 572), or tibial fractures (*n* = 638) in our hospital were enrolled from March 1999 to March 2013. According to operative procedure they underwent, the patients were evenly divided into IIN or RSIN group. Fractures were classified according to the AO classification system. The general characteristics of patients in the two groups are shown in Table 1.

**Table 1:** General information of patients with humeral, femoral or tibial fractures in two groups

**Fractures**	**Operation**	**RSIN**	**IIN**
Humerus (*n* = 494)	Age (years)	33 ± 15	32 ± 16
	Time from injury to operation (day)	6 ± 12	6 ± 14
	Causes of injury (*n*)		
	Traffic accident	140	142
	Falling injury	56	56
	Crashing injury of heavy object	35	30
	Injury caused by stroke	16	19
	Fracture type (*n*)		
	A	100	95
	B	120	113
	C	27	39
	Anterograde fixation (*n*)	137	317
	Retrograde fixation (*n*)	110	0
	Open reduction and internal fixation (*n*)	221	222
	Closed reduction and internal fixation (*n*)	26	25
Femur (*n* = 572)	Age (years)	37 ± 15	37 ± 12
	Time from injury to operation (day)	7 ± 15	7 ± 17
	Causes of injury (*n*)		
	Traffic accident	170	182
	Falling injury	86	82
	Crashing injury of heavy object	28	22
	Fracture type (*n*)		
	A	110	125
	B	97	100
	C	79	61
	Anterograde fixation (*n*)	171	174
	Retrograde fixation (*n*)	115	112
	Open reduction and internal fixation (*n*)	251	267
	Closed reduction and internal fixation (*n*)	35	19
Tibia (*n* = 638)	Age (years)	32 ± 17	32 ± 18
	Time from injury to operation (day)	8 ± 20	8 ± 20
	Causes of injury (*n*)		
	Traffic accident	185	188
	Falling injury	87	87
	Crashing injury of heavy object	45	38
	Injury caused by stroke	2	6
	Fracture type (*n*)		
	A	150	130
	B	100	115
	C	69	74
	Anterograde fixation (*n*)	319	319
	Retrograde fixation (*n*)	0	0
	Open reduction and internal fixation (*n*)	173	173
	Closed reduction and internal fixation (*n*)	146	146

*RSIN* rotary self-locking intramedullary nail; *IIN* intramedullary interlocking nail.

### RSIN design

**Figure 1:**
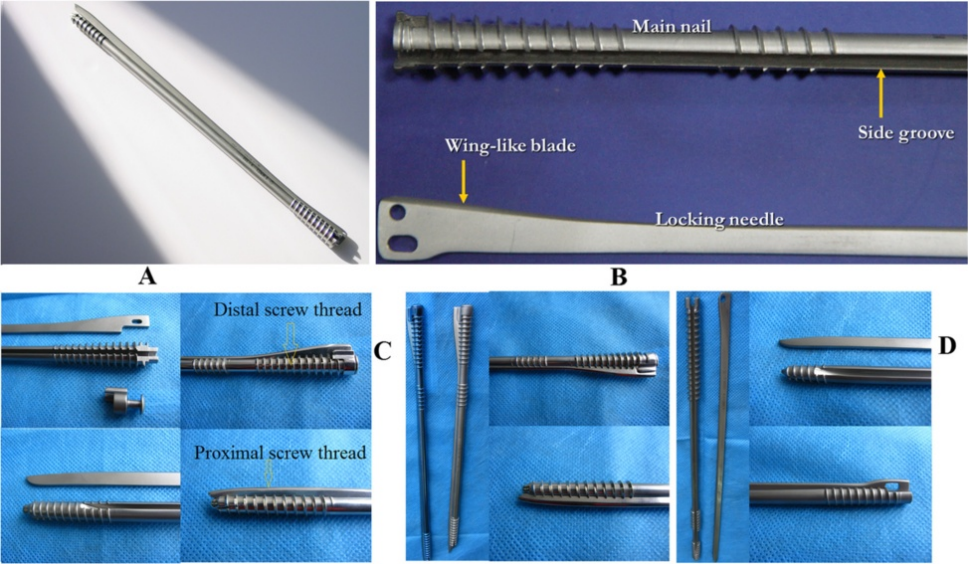
**Our self-designed rotary self-locking intramedullary nail.** The overall structure **(A)** to display the main nail and locking tag **(B)**. The nail for femoral fractures (anterograde, left; retrograde, right) **(C)**. The nail for humeral fractures (anterograde, left; retrograde, right) **(D)**.

RSIN is composed of a main rotary nail and a locking tag which are made of type 317 stainless steel or titanium alloy (Figure 1). The main nail is a round solid nail with cancellous bone screw thread at both ends. The distal screw thread is tapered, while the proximal screw thread is bullet-shaped. The screw thread pitch at proximal segment is larger than that at the distal segment. In addition, there are inner screw threads and 10-mm connection bayonet in the hollow part of the nail tail to insert the handle. There is a groove at the side of main nail to permit locking tag pass. At 20–60 mm away from the top of the main nail, the grooves gradually become shallow until they have obliquely faded out completely. The locking tag is a flat tag, whose thickness matches with the width of side groove and width is equal to groove depth plus 0.5–1 mm. The locking tag has a blade on one side along its entire length. The proximal blade is gradually widened to form a wing-like structure with a fusiform tail hole connected with a driver-extractor. Due to the anatomical differences in the femur, humerus, and tibia, our RSIN was also accordingly designed. The RSIN for humeral and femoral fractures included anterograde and retrograde nail.For femoral fractures, the anterograde nail length (Figure 1C, left) ranges from 250 to 400 mm with a diameter of 9 to 11 mm. The distal screw thread pitch is 5 mm, while the proximal is 4 mm, and the depth of screw thread is 0.4–0.8 mm; the length of thread segment at distal end and proximal end is 60–80 and 50 mm respectively; the groove is designed to allow locking tag partly pass, and the depth of groove ranges from 5 to 6 mm with a width of 3 mm. The locking tag is 10–30-mm longer than main nail, the thickness is 3 mm and the width is 5.5–6.5 mm. The retrograde nail is similar with anterograde nail except for the groove that is designed to allow the locking tag to completely pass (Figure 1C, right).For humeral fractures, the anterograde nail (Figure 1D, left) length ranges from 200 to 280 mm with a diameter of 6 to 8.5 mm. A group of screw thread was added at the tail end of main nail. The main nail is designed as all-pass groove, and other structural features are similar with anterograde nail for femoral fractures. Compared with anterograde nail, the thread segment is shorter and the thread at tail end is not tapered enlarged in retrograde nail (Figure 1D, right).

Additionally, the structure of tibia nail is similar to that of femoral nail, with the half-pass designed side groove, 280–360-mm nail length, and 8–10-mm nail diameter.

### Surgery procedure

The IIN surgery was routinely performed, while the RSIN procedure was carried out as follows.

#### ***Anterograde fixation for humeral fractures***

**Figure 2:**
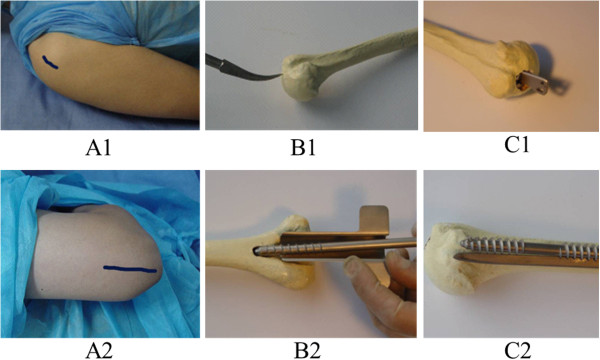
**Anterograde and retrograde fixation procedure for humeral fractures.** Anterograde fixation (*A1–C1*); retrograde fixation (*A2–C2*). *A1* incision, *B1* the entry point for the nail, *C1* main nail and locking tag screwing. *A2* incision, *B2* the entry point for the nail, *C2* the tip of main nail.

The patients were anesthetized and placed in a supine position. A 2-cm incision was made at the distal end of the acromion, and the greater tuberosity of the humerus was exposed. Mouth gag was used to drill through the medullary cavity, and the main nail was screwed into the medullary cavity for reduction under fluoroscopy. The nail tail should be located at 5 mm below the level of the greater tuberosity. Then, the locking tag was driven into the groove of main nail, ensuring that the tail of main nail and locking tag are parallel. After the intramedullary nail was implanted, the operator should touch using the finger the tag tail to ensure that there is no collision between the internal fixation and acromion. If closed reduction was difficult, open reduction and fixation was used. A small incision was made to expose the fracture segment. A 6–8-mm-diameter reamer was used to enlarge the medullary cavity to expose the greater tuberosity of the humerus and subcutaneous tissues of distal acromion. Subsequently, a 2-cm incision was made at distal acromion and the reamer pierced the subcutaneous tissues of the distal acromion. Then, the introducer was used to guide the main nail to the entry point of greater tuberosity, and the main nail was rotated into the medullary cavity to fix the fracture followed by inserting the locking tag into the groove of main nail (Figure 2 (A1, B1, C1)).

#### ***Retrograde fixation for humeral fractures***

The patient was placed in a lateral position. A 6-cm incision was made proximal to the tip of the olecranon, and the olecranon fossa was exposed after the triceps were isolated. Then, using an 8-mm drill, we drilled a hole at the posterior and midline of olecranon fossa (1.5–2 cm) along with the axis of medullary cavity. The main nail was screwed into the medullary cavity for reduction under fluoroscopy followed by retrograde screwing into proximal medullary cavity. The proximal screw thread of the main nail was screwed into cancellous bone of greater tuberosity. Also, the locking tag was driven after no shelter on the olecranon fossa was found. If closed reduction was difficult, open reduction and fixation was used (Figure 2 (A2, B2, C2)).

#### ***Anterograde fixation for femoral fractures***

**Figure 3:**
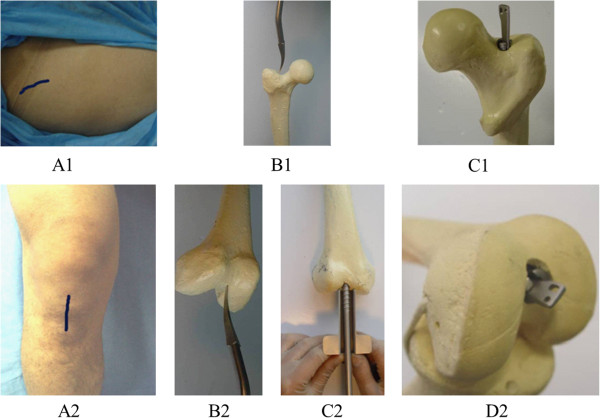
**Anterograde and retrograde fixation procedure for femoral fractures.** Anterograde fixation (*A1–C1*); retrograde fixation (*A2–C2*). *A1* incision, *B1* the entry point for the nail, *C1* main nail and locking tag screwing. *A2* incision, *B2* the entry point for the nail, *C2* main nail screwing, *D2* locking tag screwing.

The patient was left in a semi-lateral position. An 8-cm curved incision was made from 2-cm below the greater trochanter to the proximal end to expose the top of the greater trochanter. The anterior border of the trochanteric fossa was selected as the entry point of nail, and the main nail was screwed into the medullary cavity under fluoroscopy for fracture fixation following inserting of the locking tag. If closed reduction was difficult, open reduction and fixation was feasible. After the patient was positioned in a semi-lateral or lateral position, posterolateral or anterolateral approach was selected to expose the fracture segment, in which the stripping of the periosteum was reduced as much as possible. A reamer was used to enlarge the medullary cavity, and a 2-cm incision was made on the site for withdrawal of the reamer. Next, the main nail was guided into the entry point of the trochanter and screwed into the medullary cavity for fracture fixation under direct vision followed by inserting the locking tag (Figure 3 (A1, B1, C1)).

#### ***Retrograde fixation for femoral fractures***

After the patient was positioned in a horizontal position, a straight incision was made from patella to upper tibial tubercle, and ligamentum patellae were longitudinally incised. With 40-degree knee bending, the fossa intercondyloidea was exposed. A hole was drilled at 1 cm anterior to the anterior cruciate ligament and then the medullary cavity was expanded. The main nail was screwed into medullary cavity, and then the locking tag was inserted (Figure 3 (A2, B2, C2, D2)).

#### ***Anterograde fixation for tibial fractures***

**Figure 4:**
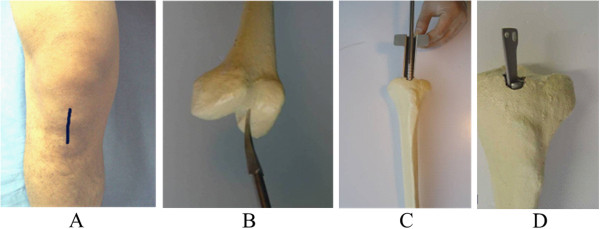
**Anterograde fixation procedures for tibial fractures. (A)** Incision; **(B)** the entry point for the nail; **(C)** main nail screwing; **(D)** locking tag screwing.

The patient lay supine after epidural anesthesia. A straight incision was made from the anterior inferior border of the patella to the upper tibial tubercle, and the ligamentum patellae were longitudinally incised. After the knee and patella were bended, a hole was drilled from the anterior border of the tibial tubercle to the medullary cavity. Following the expansion of the medullary cavity, the main nail was screwed under fluoroscopy and then locking tag was inserted. If closed reduction was difficult, open reduction and fixation was used by making a small incision. Fixation with stainless steel wire was permitted for comminuted fractures (Figure 4).

### Postoperative management

Antibiotics were used to prevent infection routinely at 3 days after the operation. The patients received functional exercise according to the postoperative status. When necessary, the continuous passive movement (CPM) was permitted after surgery. After 12 months, the internal fixation was removed after fracture healing.

Based on the different fracture positions, Neer's classification for shoulder joint [10], Aitken and Rombeck's elbow function rating system [11], Lysholm knee score [12], and the American Orthopaedic Foot and Ankle Society (AOFAS) Scoring Systems for ankle joint were used to evaluate the functional recovery of patients. The operative time, intraoperative blood loss, postoperative complications, healing rate, and the excellent rate of functional recovery for shoulder, elbow, knee, and ankle joints were recorded and compared between RSIN group and IIN group.

### Statistical analysis

The data were analyzed by using the SAS 6.2 statistical software (Version 9.1.3, SAS Institute Inc., Cary, NC, USA). All the studies were replicated with representative data shown. Measurement data were presented as mean ± standard deviation (SD), and the difference between groups was analyzed by using the Student's *t* test, while the statistical difference of enumeration data between groups was analyzed by using *χ*^2^ test. A *p* < 0.05 was considered statistically significant.

## Results

**Figure 5:**
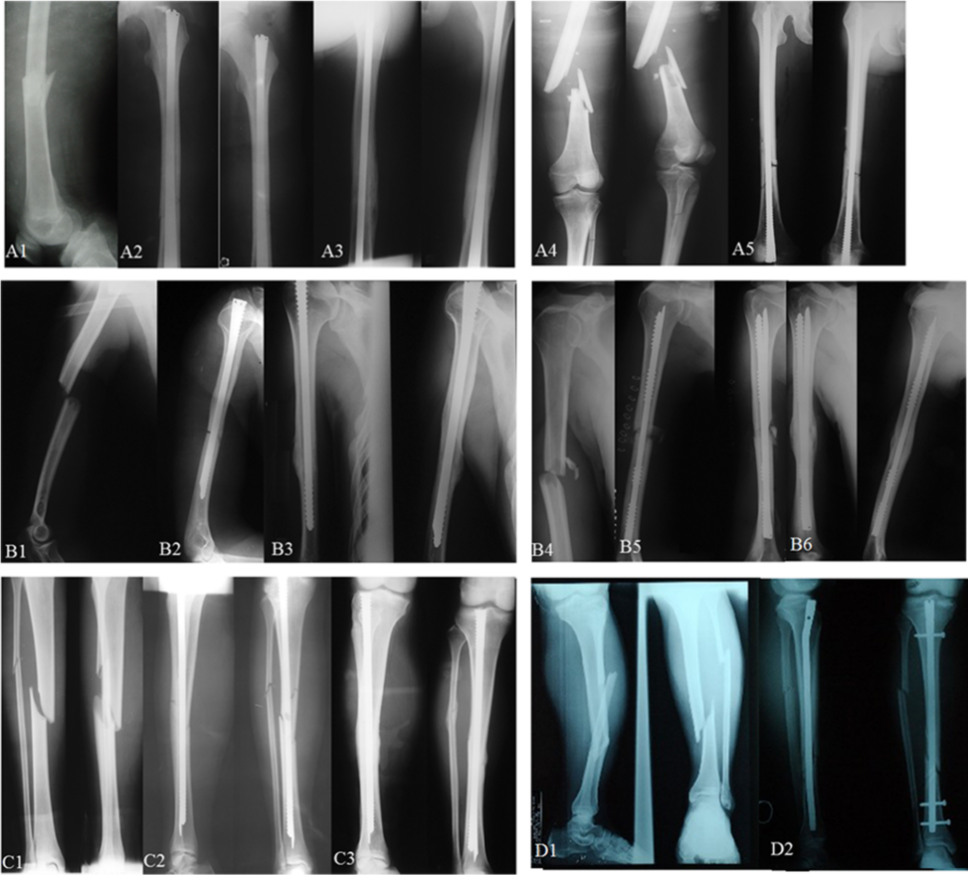
**Typical cases undergoing rotary self-locking or interlocking intramedullary nail treatment. (A–C)** rotary self-locking intramedullary nail treatment. **(A)** femoral fractures. *A1*–*A3*, anterograde and closed reduction fixation for a 53-year female patient due to traffic accident. *A1*, preoperatively; *A2*, postoperatively; *A3*, 10 months after operation bone healing. *A4*–*A5*, retrograde and open reduction fixation for a 48-year male patient due to traffic accident. *A4*, preoperatively; *A5*, postoperatively. **(B)** Humeral fractures. *B1*–*B3*, anterograde and open reduction fixation for a 28-year female patient due to falling injury. *B1*, preoperatively; *B2*, postoperatively; *B3*, 14 months after operation, bone healing. *B4*–*B6*, retrograde and open reduction fixation for a 48-year male patient due to traffic accident. *B4*, preoperatively; *B5*, postoperatively; *B6*, 12 months after operation bone healing. **(C)** Tibial fractures. *C1*–*C3*, anterograde and closed reduction fixation for a 33-year male patient due to fall injury. *C1* preoperatively; *C2* postoperatively; *C3*, 10 months after operation bone healing. **(D)** Tibial fractures undergoing interlocking intramedullary nailing. *D1*–*D2*, anterograde and open reduction fixation for a 35-year male patient due to traffic accident. *D1*, preoperatively; *D2*, postoperatively.

There were no significant differences in age, time from injury to operation, fracture type, and causes of injury of patients in the two groups, indicating it is comparable (Table 1). Compared with the IIN group, the operative time was significantly shorter, and the intraoperative blood loss was significantly lower in RSIN group no matter for humeral, femoral, or tibial fractures (all *p* < 0.001). In each group, only 317 cases were followed up for more than 1 year, including 87 humeral fractures, 104 femoral fractures, and 126 tibial fractures. The healing rate in patients with 1 year follow-up was only significantly higher in RSIN group for femoral and tibial fractures (both *p* < 0.05), but not for humeral fractures. In RSIN group, no nail breakage or loosening occurred, but radial nerve injury and incision infection were respectively observed in one patient with humeral fracture postoperatively. In IIN group, nail breakage or loosening occurred in 7 patients with femoral fractures and 16 patients with tibial fractures, radial nerve injury was observed in 8 patients with humeral fractures, and incision infection was present in 2 patients with humeral fractures and 1 patient with femoral fracture postoperatively. The complication rate of IIN group was significantly higher than that of RSIN group (*p* < 0.05). The radial nerve injury may be attributed to the distal fractures that stimulated the nerve during closed reduction or nail implantation, which was spontaneously restored at 3 months after operation. The incision infection was resolved by dressing changes. However, there were no significant differences in the excellent and good rate of shoulder, elbow, knee, and ankle joint functional recovery between RSIN group and IIN group (97.7% vs. 89.6%, shoulder and 97.7% vs. 97.7%, elbow joint for humeral fractures; 93.2% vs. 92.3%, knee joint for femoral fractures; 95.2% vs. 94.4%, knee and 95.2% vs. 94.4%, ankle joint for tibial fractures, *p* > 0.05) (Table 2). Typical cases undergoing RSIN or IIN treatment are shown in Figure 5.

**Table 2:** Intraoperative and postoperative conditions of patients with humeral, femoral or tibial fracture in two groups

**Fractures**	**Operative parameters**	**RSIN**	**IIN**	***p *** **value**
Humerus	Operative time (min)	65 ± 15	95 ± 25	<0.0001
	Intraoperative blood loss (ml)	80 ± 30	125 ± 30	<0.0001
	Healing rate in patients with 1 year follow-up (%)	95.4% (83/87)	92.0% (80/87)	0.535
	Complications (*n*)			0.046
	Radial nerve injury	1	8	
	Incision infection	1	2	
	Neer score (*n*)			0.092
	Excellent	79	73	
	Good	6	5	
	Poor	2	9	
	Aitken and Rombeck's score (*n*)			0.707
	Excellent	74	73	
	Good	11	12	
	Poor	2	4	
Femur	Operative time (min)	70 ± 20	100 ± 30	<0.0001
	Intraoperative blood loss (ml)	150 ± 50	190 ± 60	<0.0001
	Healing rate in patients with one year follow up (%)	98.1% (102/104)	88.5% (92/104)	0.010
	Complications (*n*)			0.016
	Incision infection	0	1	
	Nail breakage or loosening	0	7	
	Lysholm score (*n*)			0.992
	Excellent	84	83	
	Good	13	13	
	Fair	5	6	
	Poor	2	2	
Tibia	Operative time (min)	55 ± 15	85 ± 25	<0.0001
	Intraoperative blood loss (ml)	10 ± 5	15 ± 10	<0.0001
	Healing rate in patients with 1 year follow up	92.1% (116/126)	81.2% (103/126)	0.024
	Complications (*n*)			0.000
	Nail breakage or loosening	0	16	
	Lysholm score (*n*)			0.986
	Excellent	110	109	
	Good	10	10	
	Fair	3	4	
	Poor	3	3	
	AOFAS score (*n*)			0.990
	Excellent	113	112	
	Good	7	7	
	Fair	4	5	
	Poor	2	2	

*RSIN* rotary self-locking intramedullary nail, *IIN* intramedullary interlocking nail, *AOFAS* American Orthopaedic Foot and Ankle Society.

## Discussions

In the present study, we demonstrated that our self-designed RSIN may be a reliable and practical alternative method for the treatment of long bone fractures because the RSIN group exhibited significantly shorter operative time, less intraoperative blood loss, higher healing rate, and excellent and good rate of shoulder, elbow, knee, and ankle joint functional recovery and few complications compared with IIN group.

The superior results may be attributed to the specific design of our RSIN:

(1) By making only one small incision, the main nail is rotated into the medullary cavity via the cancellous bone screw thread at both of its ends, thus reducing the need for excessive reaming. It is reported that the excessive reaming of the medullary cavity causes an increase in intramedullary pressure, which provokes intravasation of the bone marrow and fat into the venous blood system, leading to intravascular thrombosis and adult respiratory distress syndrome [13]. Moreover, excessive cortical reaming also generates significant heat and induces cortical thermal necrosis, influencing bone healing [14,15], while limited reaming may improve blood flow in the surrounding soft tissues and promote bone formation [16]. As expected, the healing rate of our study was significantly higher in RSIN group.

(2) The locking tag can be inserted into the medullary cavity along the side groove of the main nail not requiring an aiming device and X-ray localization to guide [17]. Thus, the operation is relatively simple and less time-consuming, which was also demonstrated in our study (65 ± 15 min vs. 95 ± 25 min for humeral fractures; 70 ± 20 min vs. 100 ± 30 min for femoral fractures; 55 ± 15 min vs. 85 ± 25 min for tibial fractures; all *p* < 0.001).

(3) The different screw thread pitch of main nail at proximal and distal segment plays a limited compression role. The proximal segment of locking tag is embedded into the proximal bone of the fracture and its distal segment bifurcates laterally along the groove to be embedded into the distal cortex of the fracture. This longitudinal, filled, locking fixation realizes bidirectional self-locking to maintain the roles of anti-rotation and anti-contraction [18]. The advantage of this fixation model is a relatively static fixation at early stage but relatively dynamic fixation at the middle and later periods due to local bone absorption and muscle contraction, which avoids stress concentration to induce nail breakage [19]. In line with the above theory, no nail breakage was observed in our study.

However, some postoperative complications occurred using our RSIN for the treatment of humeral fractures, including radial nerve injury and incision infection. Nerve injury is a common complication after operative exposure and fixation of humeral shaft fractures. Although various nerves in the arm may be involved, the radial nerve is one of the most commonly involved. The radial nerve is located at the lateral intermuscular septum within the distal third of the humerus, where the range of motion is small. Surgical dissection in this region will cause tractional damage to this nerve [20]. Recent studies report that the rate of iatrogenic radial nerve injury in operatively treated humeral fractures is 4%–8% [21,22]. However, only one patient developed radial nerve injury in RSIN group, and he was spontaneously restored at 3 months after the operation. Thus, further attention should be paid during RSIN fixation.

Surgical indications for our RSIN are as follows: (1) humeral fractures: this procedure is applicable to shaft fracture located at 3 cm below the greater tuberosity of the humerus and 5 cm over the olecranon fossa but not the serious comminuted fractures or fractures with the osteoporosis at proximal end; (2) femoral fractures: this procedure is applicable to fractures located below the greater trochanter to 8 cm over the supracondylar segment but not the serious comminuted fractures; (3) tibial fractures: this procedure is applicable to fractures at the upper 1/3 of tibia and 8 cm over the supracondylar segment.

In conclusion, our self-designed RSIN may be an effective and safe approach for treating long bone fractures compared with IIN. However, further studies with large sample size and longer follow-up are still needed to comprehensively evaluate our RSIN.

## Competing interests

The authors declare that they have no competing interests.

## Authors’ contributions

BLL and YX conceived and designed the research; HD, SG, FJ, and QHL analyzed the data; DXW contributed reagents/materials/analysis tools; XWG and WL wrote the paper. All authors read and approved the final manuscript.

## Authors’ information

Bailian Liu and Ying Xiong joint first authors.
